# Lactic acid regulates lipid droplet aggregation through a microglia-neuron axis in neuroinflammation

**DOI:** 10.1016/j.jlr.2024.100629

**Published:** 2024-08-23

**Authors:** Zhuoqing Lan, Shukai Lv, Ziyi Ge, Bing Zhao, Leilei Li, Caixia Li

**Affiliations:** 1 Department of Anesthesiology, First Affiliated Hospital, School of Medicine, Zhejiang University, Hangzhou, China; 2 Fourth Affiliated Hospital, School of Medicine, Zhejiang University, Yiwu, China; 3 Department of General Medicine, Zhejiang Provincial People's Hospital, Hangzhou, China

**Keywords:** microglia, neuron, lipid droplet, lactic acid, RNA sequencing, neuroinflammation

## Abstract

Neuroinflammation, marked by the release of proinflammatory cytokines and resulting neuronal death, is a multifaceted process extending beyond traditional inflammatory pathways. Microglia, primary cells in the inflammatory response, rapidly activate during neuroinflammation and produce proinflammatory and cytotoxic factors that affect neuronal function. Recent evidence highlights the significant role of abnormal lipid droplet (LD) deposition in the pathogenesis of neuroinflammation. While microglia are known to influence LD aggregation during neuroinflammation, the regulatory mechanism within neurons is not well understood. Our study demonstrates that lipopolysaccharide-activated microglia induce the accumulation of LD in neurons, identifying microglial-derived lactic acid as a key mediator in this process. Excessive lipid accumulation threatens neuronal function, a phenomenon reversed by eliminating microglia. Our study demonstrates that lipopolysaccharide-activated microglia induce the accumulation of LD in neurons, identifying microglial-derived lactic acid as a key mediator in this process. Excessive lipid accumulation threatens neuronal function, a phenomenon reversed by eliminating microglia.

Neuroinflammation, characterized by an inflammatory response in the central nervous system (CNS), plays a pivotal role in various disease states (1), including ischemic hypoxic encephalopathy, stroke, and neurodegenerative diseases like Parkinson's disease and Alzheimer's disease (1, 2). A key feature of neuroinflammation is microglia activation, which leads to the release of proinflammatory factors and extensive transcriptional changes in metabolic-related genes. Proinflammatory stimuli can shift microglial metabolism from oxidative phosphorylation to glycolysis, a transition crucial for regulating immune functions during acute neuroinflammation (3).

In a physiological state, microglia are in a quiescent state, maintaining CNS homeostasis by secreting various anti-inflammatory factors and signaling molecules (4). Upon activation, microglia undergo morphological and functional changes (5, 6). Targeting metabolism in microglia-mediated neuroinflammation has significant potential. Microglia undergo metabolic reprogramming, shifting from oxidative phosphorylation to glycolysis and the pentose phosphate pathway, impacting glucose and lipid metabolism (6, 7). However, the impact of this metabolic reprogramming on neurons and its specific regulatory mechanisms remains unclear.

Upon lipopolysaccharide (LPS) stimulation, microglia not only produce lactate through glycolysis but can also import it from the extracellular space, as shown in vitro (6, 8). Lactate serves as a key metabolite and signaling molecule, supporting energy demands and influencing cellular functions (9, 10). In addition, LPS-activated microglia exhibit increased expression of the lactate transporter monocarboxylate transporter 1 (MCT 1) (6). Lactate accumulation is observed in various acute and chronic neuroinflammatory conditions, including ischemic stroke, neurodegenerative diseases, and aging (11, 12, 13).

In addition to lactate, lipid droplet (LD) also plays a role in microglia-neuron metabolic interactions. LD are organelles that store neutral lipids and have a unique structure composed of phospholipid monolayer surrounding a hydrophobic core of triacylglycerols (TAGs) and cholesteryl esters (CEs) (14). CEs are vital for maintaining cell membrane integrity and fluidity and are crucial for neuronal synapse and dendrite formation (15, 16, 17). Dysregulation of cholesterol metabolism can often lead to structural and functional CNS diseases, such as Niemann-Pick disease, Huntington’s disease, Alzheimer's disease, and Parkinson's disease (18, 19, 20). Excessive CE accumulation can regulate inflammation, and aging via mechanistic target of rapamycin complex 1 activation and induce intracellular oxidative stress, mitochondrial damage, and ultimately apoptosis (21, 22).

This study aimed to determine the detrimental role of activated microglia in neuroinflammation, focusing on abnormal LD aggregation in neurons. We identified microglial-derived lactic acid as a key mediator of this process, highlighting its potential as a therapeutic target for acute neuroinflammation. Furthermore, we also assessed the impact of lipid accumulation on pyroptotic neuronal cell death and cell viability during acute neuroinflammation. Our results were validated in a neuroinflammatory mouse model, and RNA sequencing (RNA-seq) was employed to analyze lipid changes, providing insights into potential target mechanisms for future exploration.

## Materials and Methods

### Animal experimentation

Male C57BL/6 mice (6–8 weeks old, 18–20 g) were purchased from the Experimental Animal Center, Zhejiang Academy of Medicine Sciences, Hangzhou, China (Certificate No. SYXK Zhe 2019-0011). Mice were housed at 22 ± 2°C, relative humidity 50 ± 10%, and a 12-h light/dark cycle. They were allowed to have free access to water and food. All experiments followed the National Institutes of Health Guide for the Care and Use of Laboratory Animals. The number of animals used in the study and their suffering were minimized. The experimental protocols were approved by the Ethics Committee of Laboratory Animal Care and Welfare, School of Medicine, Zhejiang University, Hangzhou, China.

### PLX 3397 treatment

For pharmacological ablation of microglia in mouse gavage experiments, a working solution of pexidartinib (PLX3397, GLPBIO, CA) was made in 10% DMSO (Solarbio.BeiJing, China) and 40% PEG300 (Solarbio.BeiJing, China) in water to obtain a final concentration of 10 mg/ml. Mice were administered with PLX3397 via oral gavage at a dosage of 80 mg/kg/day every day for three consecutive weeks, as described previously (formulated in AIN-76A standard irradiated chow by Research Diets at a dose of 290 mg/kg for PLX3397) (23). Mice in the control group were treated with the same volume of vehicle (normal saline).

### The neuroinflammation mouse model

The neuroinflammation mouse model was established as described previously, that is, via intraperitoneal injection of 1 mg/kg LPS solution for seven consecutive days. The control group received injections of an equivalent volume of physiological saline, adjusted based on body mass. The neurobehavioral assessment was determined by a modified neurologic severity score (24). The neuroinflammation mouse model was defined as having a score ≥7.

### Experimental groups

Mice were randomly divided into four groups (each consisting of 20 mice): sham (vehicle), LPS, vehicle +PLX3397, and LPS+PLX3397. Mice in the four groups were treated with either vehicle or PLX3397 by oral gavage. On the 14th day, mice were injected with LPS for the neuroinflammation procedure. At the end of the experiment (day 21), mice were reanesthetized and perfused with saline, followed by 4% paraformaldehyde (PFA). Brains were removed, fixed in 4% PFA for 24 h, and transferred to a 30% sucrose solution for three days. Next, 20-μm sections from the frontal to the occipital poles were cut by cryomicrotome (Leica CM1950, Wetzlar, Germany) and were further used for immunostaining. The brains were collected and stored at −80°C for biochemical analysis and immunoblotting.

### Primary cortical neuron culture

Primary hippocampal neurons were obtained from the cortex of neonatal Sprague-Dawley rats (Experimental Animal Center, Zhejiang Academy of Medicine Sciences, Hangzhou, China). Briefly, the cortex of neonatal Sprague-Dawley rats was dissected and digested with 0.25% EDTA-free trypsin at 37°C for 10 min. The dissociated cells were immediately seeded onto poly-l-lysine precoated flasks and incubated in high glucose DMEM containing 10% fetal bovine serum , 10% horse serum, 2 mM glutamine, 100 μg/ml streptomycin, and 100 U/ml penicillin for 24 h in a humidified atmosphere of 5% CO_2_/95% air at 37°C (standard cell culture conditions). After 24 h, the medium was changed to high glucose DMEM containing 5% horse serum, 2 mM glutamine, 100 U/ml penicillin, 100 μg/ml streptomycin, 0.04% B27, and 0.01% N2. On day 3, 10 μM cytosine arabinoside was added for 24 h to inhibit the growth of glial cells in cultured neurons; the medium was refreshed every three days. On day 10, >95% of the cultured cells were neurons, as determined by immunofluorescent staining for microtubule-associated protein-2 (MAP-2).

### Cell culture

Mouse BV2 microglial cells, murine microglia N9 cells, mouse HT-22 hippocampal neuron cells, and human SH-SY5Y neuroblastoma cells were obtained from the Chinese Academy of Medical Sciences (Beijing, China). BV2, N9, and HT-22 cells were cultured in RPMI-1640 medium supplemented with 10% FBS, 5 U/ml penicillin, and 5 μg/ml streptomycin under standard cell culture conditions. SH-SY5Y line was cultured in 43% Ham’s F-12 medium and 43% MEM basal medium supplemented with 10% FBS, 1% MEM non-essential amino acids, 1% sodium pyruvate, 1% GlutaMAX-1, 5 U/ml penicillin, and 5 μg/ml streptomycin under standard cell culture conditions. To avoid activating microglia, FBS was heat inactivated for 30 min at 56°C before use. Before stimulation, cells were seeded in 5 × 10ˆ5 cells per well in a tissue culture-treated 6-well plate (Corning, NYC) and grown to 80% confluency. For the experiments, BV2 and N9 cells were pretreated with 1 μg/ml LPS (Sigma-Aldrich, MO) for 18 h to stimulate LD formation. The cells were treated with Toll-like receptor 4 (TLR4) inhibitor 1 uM/L TAK-242 (GLPBIO, MFEE).

### Conditioned medium treatment

To evaluate the effects of the microglial conditioned medium (CM) on primary neurons, HT-22 or SH-SY5Y cells, the supernatant was collected from the BV2 and N9 cells pretreated with 1 μg/ml LPS. The CM was collected and centrifuged at 6,000 rpm at 4°C for 10 min to remove cell debris. Primary hippocampal neurons, HT-22, or SH-SY5Y cells were incubated with the CM from the microglia for 18 h. Subsequently, cell viability and pyroptosis were assessed.

### Transwell coculture system

RPMI-1640 supplemented with 10% FBS, 5 U/ml penicillin, and 5 μg/ml streptomycin was used as a medium. To avoid activating BV2 and N9 cells, FBS was heat inactivated for 30 min at 56°C before use. HT-22 cells plated on a coverslip were transferred into the lower compartment of a 6-well transwell system, and BV2 microglia were plated (10^6^ cells per well) onto the insert of the transwell (0.4 mm pore size polycarbonate membrane precoated with poly-l-lysine).

### Cell viability determination

Cell viability was determined using cell counting kit-8 (CCK-8; Beyotime Technology Inc., Shanghai, China) according to the manufacturer's instructions. Cells were seeded into a 96-well plate and treated with CM in the presence or absence of LPS. After treatment, the cells were rinsed with PBS and incubated with 10 μl cell counting kit-8 solution for 1.5 h at 37°C. The absorbance was measured at 450 nm using a microplate reader (Bio-Rad, Hercules, CA). Data were expressed as % of controls.

Cell viability was also determined using methylthiazolyldiphenyl-tetrazolium bromide assay (Solarbio Technology Inc., Beijing, China) according to the manufacturer's instructions. Cells were seeded into a 96-well plate and treated with CM in the presence or absence of LPS. After treatment, remove the supernatant carefully, add 90 μl culture solution, then add 10 μl methylthiazolyldiphenyl-tetrazolium bromide solution, and continue to culture for 4 h. Then absorb the supernatant, add 110 μl of Formazan solution to each hole, and oscillate at a low speed for 10 min on a shaking table to fully dissolve the crystal. Absorbance was measured at 490 nm using a microplate reader (Bio-Rad, Hercules, CA). Data were expressed as % of controls.

### Immunostaining

Immunostaining was performed to determine the localization of LDs in different cell types. The 20-μm brain sections were rinsed with 0.01 M PBS, incubated for 1 h with 5% goat serum (Zhongshan Biotechnology Co, Beijing, China) supplemented with 0.3% Triton X-100 to block nonspecific immunoglobulin (Ig) G binding, and then incubated overnight at 4°C with the following primary antibodies: rabbit anti-MAP-2 (1:200; Affinity Biosciences), mouse anti-neuronal nuclear antigen (NeuN, 1:500, Millipore, Billerica, MA), rabbit anti-ionized calcium-binding adapter molecule 1 (IBA1, 1:500, Wako Pure Chemical Industries, Ltd.), mouse anti-glial fibrillary acidic protein (GFAP, 1:800, Millipore, Billerica, MA), mouse anti-cleaved caspase 1 (1:200, Affinity Biosciences), and mouse anti-caspase 1 (1:200, Affinity Biosciences). Sections were then incubated with DyLight 549– or DyLight 488–conjugated secondary antibody (1:200, EarthOx SF) for 2 h at room temperature. Nuclei were stained with 4',6-diamidino-2-phenylindole (DAPI).

Cells seeded on coverslips in 24-well plates were fixed in 4% PFA for 20 min, permeabilized with 0.5% Triton X-100 for 10 min, and blocked with 5% goat serum for 30 min at 37°C. Cells were treated overnight at 4°C with the following primary antibodies: anti-MAP-2 (1:200), anti-cleaved caspase 1 (1:200), and anti-caspase 1 (1:200), followed by DyLight 549– or DyLight 488–conjugated secondary antibody (1:200) for 2 h at room temperature. For the negative control, PBS was used instead of the primary antibody. Nuclei were stained with DAPI. After mounting with an Anti-Fade Mounting Medium (Beyotime Technology Inc., Shanghai, China), images were taken with the Olympus IX71 fluorescence microscope equipped with a DP71 digital camera using Ocular software. Eight images of nonoverlapping fields for each site in the same sections (40x magnification) were randomly taken, and the average area of LDs (μm^2^) per view field and the average number of LDs per cell were calculated using ImageJ software.

### BODIPY staining

4,4-difluoro-1,3,5,7,8-pentamethyl-4-bora-3a,4a-diaza-s-indacene (BODIPY 493/503, Invitrogen; Thermo Fisher Scientific, Inc.) fluorescent neutral lipid dye was used to visualize LDs in cells or brain sections by fluorescent microscopy. The 20-μm brain sections or fixed cells on coverslips in 6- or 24-well plates were rinsed with 0.01 M PBS and then incubated for 30 min with BODIPY 493/503 at 37°C. After mounting with an anti-fade medium, the stained samples were examined under the Olympus IX71 fluorescence microscope. Eight images of nonoverlapping fields for each site in the same sample (40x magnification) were randomly taken, and Image J software was used to determine the per-cell number of lipid vesicles for each view field.

### Immunoblotting

The brain tissue or total cell protein extraction was performed using ice-cold RIPA buffer (Beyotime Technology Inc., Shanghai, China) according to the manufacturer's instructions. The homogenates were centrifuged at 12,000 *g* at 4°C for 30 min, and the supernatant was used for subsequent analyses. Equal amounts of protein (60 μg) for each sample were separated by 15% SDS-PAGE and transferred to a PVDF membrane. The membranes were blocked in 5% BSA and sequentially incubated with primary antibodies: anti-IBA1 (1:500), anti-cleaved caspase 1 (1:200), and mouse anti-GAPDH (1:5000, Kang Chen Technology Inc., Shanghai, China). Next, the membranes were incubated with HRP-conjugated goat anti-rabbit IgG or goat anti-mouse IgG (1:200, Thermo Fisher Scientific) for 2 h at room temperature. Proteins were visualized using chemiluminescent reagents according to the manufacturer’s instructions (Merck, Millipore) with a Bio-Rad ChemiDoc Touch Imaging System and quantified with Quantity One software (Bio-Rad, Hercules, CA).

### Assessment of cell pyroptosis

Cell pyroptosis was measured using the FLICA 660 Caspase-1 Kit (MyBioSource, VAN, CAN) according to the manufacturer’s instructions (25). Briefly, after 18 h treatment with LPS, cells were collected, washed once with cold PBS, and centrifuged at 2,000 *g* for 5 min. The pellet was resuspended in 300 μl of binding buffer containing active caspase 1-FITC and propidium iodide solution and then stained in the dark for 15 min at room temperature. The percentage of cell pyroptosis was determined using a FACSCalibur cytometer (Beckman Coulter CytoFLEX S, CA). The fold increase in cell death rate was normalized to that of the control group.

### Measurement of lactic acid, triglyceride, and cholesterol levels

Lactic acid, triglyceride (TG), and cholesterol levels were measured according to the manufacturer’s instructions (TG assay kit, total cholesterol assay kit, lactic acid assay kit; Jiancheng Technology Inc., Nanjing, China). After 18 h of stimulation with CM, the brain tissue homogenates, cultured cell supernatants, or cell lysates were collected and used for the subsequent analysis of lactic acid, TG, or cholesterol concentrations. The values were calculated by measuring the absorbance at 500 nm (CE), 510 nm (TAG), and 530 nm (lactic acid) using a multifunctional enzyme standard in a multimode plate reader (Tecan Spark, Tecan, Switzerland).

### RNA isolation and library preparation

Total RNA was extracted using the TRIzol reagent (Invitrogen, CA) according to the manufacturer’s protocol. RNA purity and quantification were assessed using the NanoDrop 2000 spectrophotometer (Thermo Fisher Scientific). RNA integrity was evaluated with the Agilent 2100 Bioanalyzer (Agilent Technologies, Santa Clara, CA). Subsequently, libraries were constructed using VAHTS Universal V6 RNA-seq Library Prep Kit following the manufacturer’s instructions. The transcriptome sequencing and analysis were conducted by OE Biotech Co., Ltd. (Shanghai, China).

### RNA-seq and differentially expressed genes analysis

The libraries were sequenced on an Illumina Novaseq 6000 platform, generating 150 bp paired-end reads. Raw reads in fastq format were initially processed using fastp to eliminate low-quality reads, resulting in clean reads. HISAT was employed to map the clean reads to the reference genome. Fragments per kilobase of exon model per million mapped fragments for each gene was calculated, and read counts were obtained by HTSeq-count. Principal component analysis analysis was performed in R (v 3.2.0) to evaluate biological sample duplication. Differential expression analysis was performed using DESeq2, with a threshold set at Q value < 0.05 and foldchange > 2 or foldchange < 0.5 to identify significantly differentially expressed genes (DEGs). Hierarchical cluster analysis of DEGs was conducted using R (v 3.2.0) to illustrate gene expression patterns across different groups and samples. Based on the hypergeometric distribution, Gene Ontology (GO), Kyoto Encyclopedia of Genes and Genomes pathway, reactome, and WikiPathways enrichment analyses of DEGs were performed using R (v 3.2.0) to identify significantly enriched terms.

### Statistical analysis

All data were expressed as means ± SEM. Student's *t* test was used to compare statistical differences between two groups, and one-way ANOVA followed by Tukey's test was applied for multiple comparisons. *P* < 0.05 was considered a statistically significant difference.

## Results

### Activated microglia induce LD aggregation in neurons

**Fig. 1:**
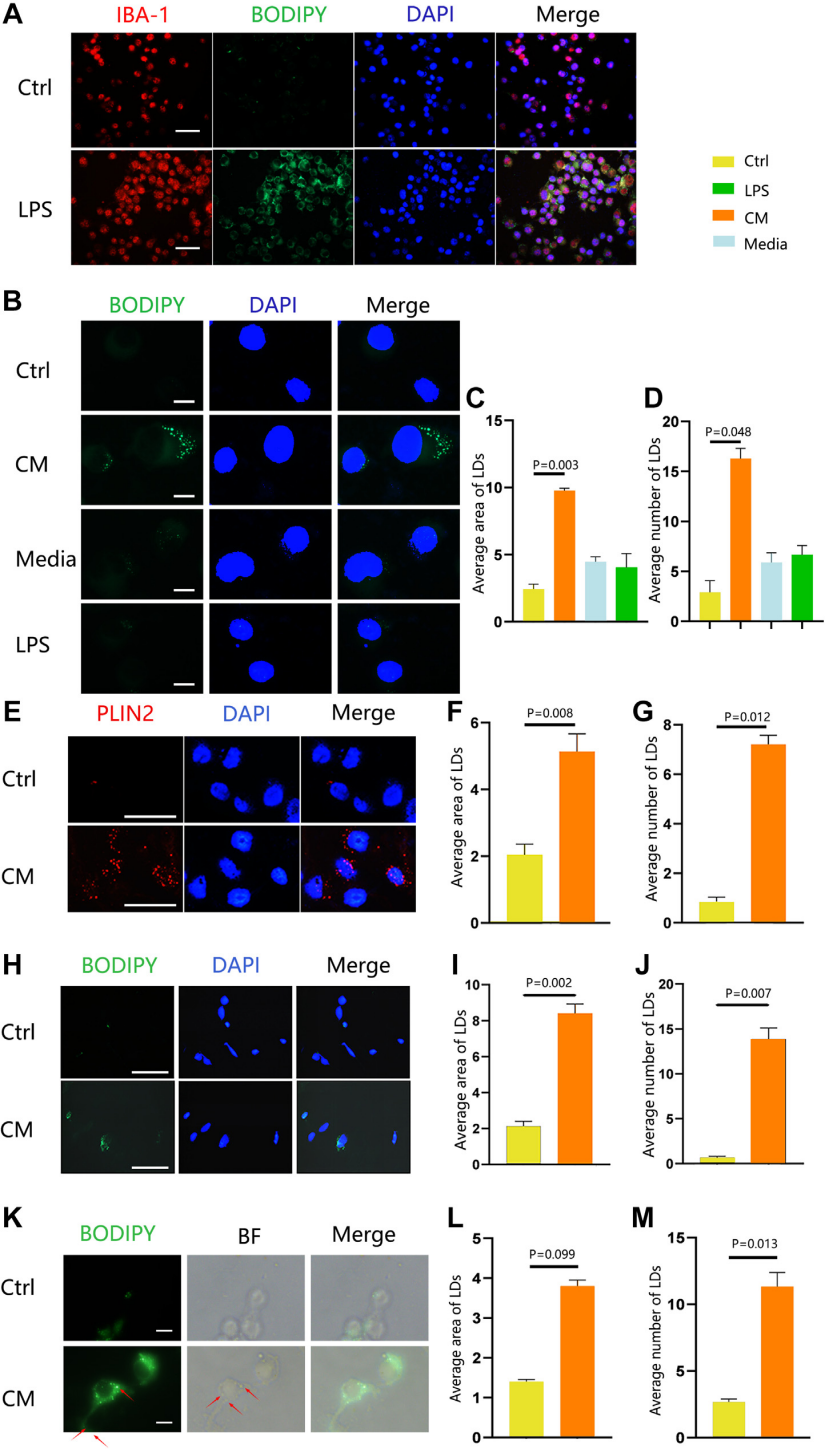
Formation of LDs in neurons induced by the supernatant from the activated microglia. (A) Representative fluorescent images of LD staining in BV2 cells treated with 1 μg/ml LPS for 18 h. BODIPY was used to visualize LDs; microglia were stained with IBA-1 and nuclei with DAPI. (B–D) HT-22 cells treated with a culture supernatant of PBS-stimulated microglia (Ctrl), LPS, or CM. (B) Representative fluorescent images of LDs visualized with BODIPY. (C) Average area of LDs (μm^2^) and (D) average number of LDs (per cell). (E–G) Representative fluorescent images of PLIN2 staining in HT-22 cells treated with Ctrl or CM for 18 h. (E) PLIN2 was used to visualize LDs, and nuclei were stained with DAPI. (F) Average area of LDs (μm^2^) and (G) average number of LDs (per cell). (H–J) Representative fluorescent images of LD staining in LPS-stimulated SY5Y cells. (H) BODIPY was used to visualize LDs. (I) Average area of LDs (μm2) and (J) average number of LDs (per cell). Values are mean ± SEM, n = 3. ∗*P* < 0.05; ∗∗*P* < 0.01. Scale bars: 10 μm. (K–M) Representative fluorescent images of LD staining in LPS-stimulated N9 cells. (K) BODIPY was used to visualize LDs. (L) Average area of LDs (μm2) and (M) average number of LDs (per cell). Values are mean ± SEM, n = 3. ∗*P* < 0.05; ∗∗*P* < 0.01. Scale bars: 5 μm. CM, conditioned medium; DAPI, 4',6-diamidino-2-phenylindole; IBA1, ionized calcium-binding adapter molecule; LD, lipid droplet; LPS, lipopolysaccharide.

Previous studies demonstrated LD accumulation in microglia following LPS exposure (26), and altered transcriptomes suggested that LPS treatment mimics microglial activation and the inflammatory response observed in neuroinflammation (27). In our study, microglia (BV2 cells) stimulated with LPS for 18 h exhibited an increase in LDs number, as indicated by BODIPY staining (Fig. 1A). To investigate whether activated microglia can regulate neuronal lipid metabolism in a non–cell-autonomous fashion, neurons were incubated in conditioned media derived from microglia cultured in basal conditions or LPS-activated CM. This resulted in an evident increase in LDs area and number per cell compared to the control and LPS groups (Fig. 1B–D). Perilipins (e.g., PLIN2), proteins involved in lipid metabolism associated with the phospholipid monolayer surrounding LDs (28), were found to colocalize with LDs in neurons, supporting our findings (Fig. 1E–G). Validation in SY5Y cells treated with CM further supported LD aggregation (Fig. 1H–J). Additionally, we have successfully replicated these results in the N9 microglial cell line (Fig. 1K–M).

**Fig. 2:**
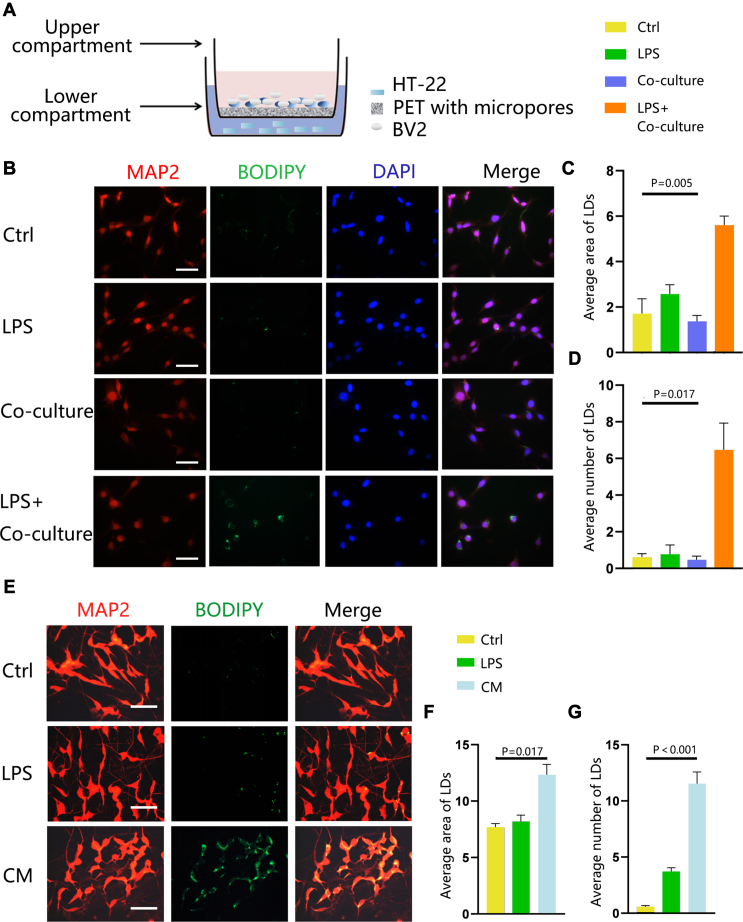
Activated microglia cause neuronal LD aggregation in a co-culture system and primary rat cortical neurons. (A) Schematic diagram of the coculture system. (B–D) CM-induced aggregation of LDs in HT22 cells was studied in the transwell coculture system. (B) Representative fluorescent images of LDs. BODIPY was used to visualize LDs; neurons were stained with MAP-2 and nuclei with DAPI. Ctrl represents PBS-stimulated neurons, LPS represents LPS-stimulated neurons, and co-culture represents transwell coculture system. (C) Average area of LDs (μm^2^) and (D) average number of LDs (per cell). The LPS + coculture group was compared with other experimental groups. (E–G) The primary rat cortical neurons were treated with PBS (Ctrl), 1 μg/ml LPS, or CM for 18 h. (E) Representative fluorescent images of LDs. BODIPY was used to visualize LDs, and neurons were stained with MAP-2. (F) Average area of LDs (μm^2^), and (G) average number of LDs (per cell). Values are mean ± SEM, n = 3. ∗*P* < 0.05; ∗∗*P* < 0.01. Scale bars: 10 μm. CM, conditioned medium; DAPI, 4',6-diamidino-2-phenylindole; LD, lipid droplet; LPS, lipopolysaccharide; MAP-2, microtubule-associated protein-2.

**Fig. 3:**
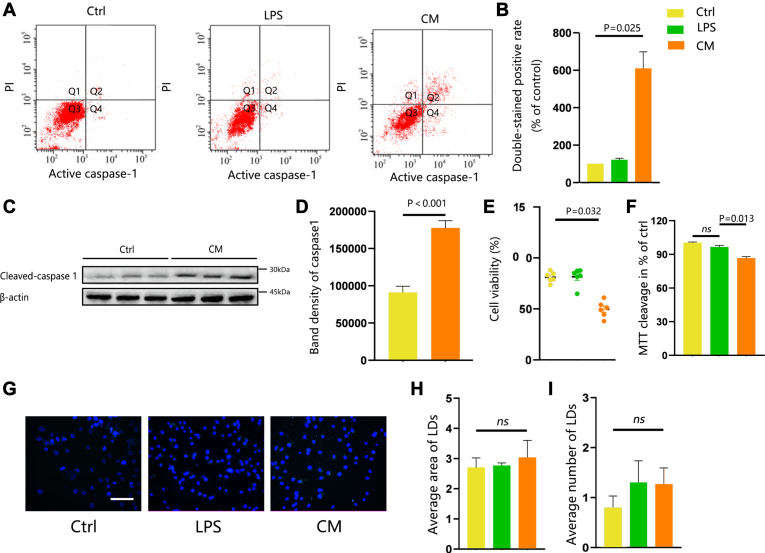
Accumulated LDs impair neuronal function. (A–D) HT-22 cells treated with CM compared to Ctrl or LPS. (A) Representative flow cytometry dot plots showing pyroptosis ratio based on the differences in the Q2 quadrant. (B) The percentage of active caspase-1 and PI double-stained positive cells normalized to the Ctrl group. (C) Representative Western blot images of levels of cleaved caspase1. β-actin was used as a loading control. (D) Band density of caspase1 represented as values quantified from protein bands of caspase1 normalized to β-actin. (E, F) Cell viability assessment in HT-22 cell line. (G–I) Representative fluorescent images of LD staining with BODIPY in astrocytes treated with CM and Ctrl. (G) BODIPY was used to visualize LDs; astrocytes were stained with MAP-2 and nuclei with DAPI. (H) Average area of LDs (μm2) and (I) average number of LDs (per cell). Values are mean ± SEM, n = 3. ∗*P* < 0.05; ∗∗*P* < 0.01, ∗∗∗*P* < 0.001. Scale bars: 10 μm. CM, conditioned medium; DAPI, 4',6-diamidino-2-phenylindole; LD, lipid droplet; LPS, lipopolysaccharide; MAP-2, microtubule-associated protein-2.

Experiments were repeated using the transwell chambers and primary rat cortical neurons to closely mimic the complex physiological environment. The specific setup of the transwell coculture system is shown in Fig. 2A. LD accumulation in neurons upon LPS stimulation in coculture was observed (Fig. 2B–D). Validation in primary neurons treated with CM showed increased LD number and area compared to the control group (Fig. 2F, G). Subsequently, the impact of LD aggregation on neurons was explored by assessing cell viability and pyroptosis. An increase in pyroptosis (caspase-1–dependent programmed cell death (Fig. 3A–D) and a decrease in cell viability (Fig. 3E, F) were observed, indicating that LD accumulation is detrimental to neurons. Notably, activated astrocytes did not induce LD accumulation in neurons (Fig. 3G–I), supporting the fact that activated microglia specifically induce LD aggregation in neurons during neuroinflammation.

### LDs accumulated in neurons do not originate from microglia

**Fig. 4:**
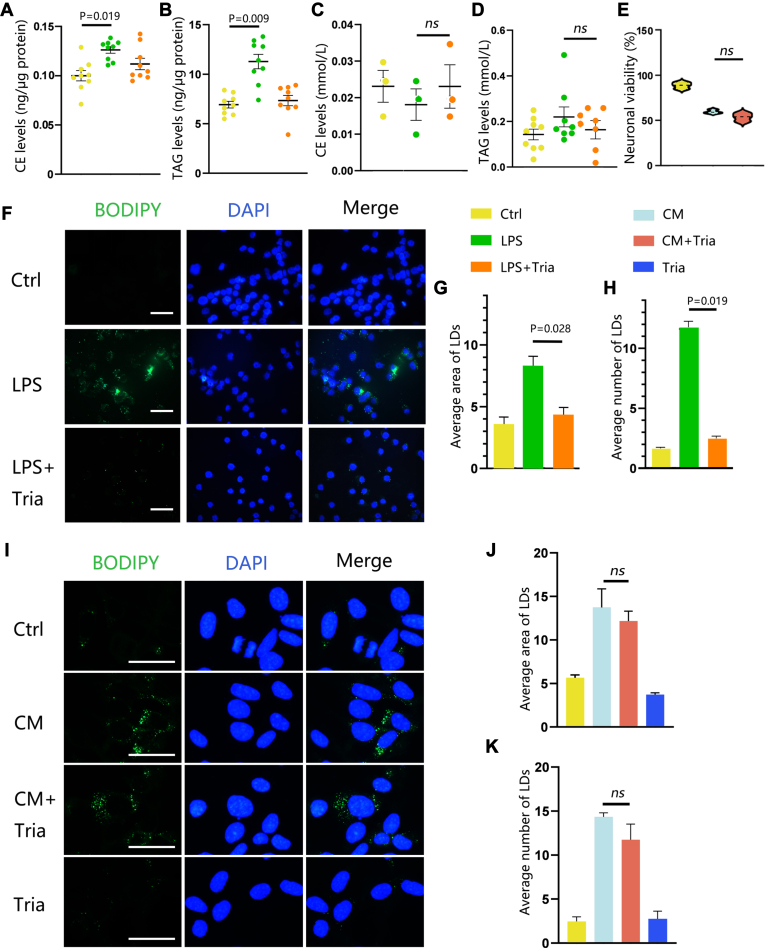
LDs accumulated in neurons do not come from microglia. (A) The level of cholesteryl ester (CE) and (B) triacylglycerols (TAGs) in microglia. (C) The levels of CE and (D) TAG in microglia supernatant. (E) Inhibition of the lipid synthesis (triacsin C, Tria) in CM cannot reverse neuronal viability (CM group compared to CM+Tria group). (F–H) BV2 cells were treated with PBS (Ctrl), 1 μg/ml LPS, and 1 μg/ml LPS with 1 μM Tria for 18 h. (F) Representative fluorescent images of LDs visualized with BODIPY. Scale bars: 5 μm. (G) Average area of LDs (μm^2^) and (H) average number of LDs (per cell) in BV2 cells. The LPS group was compared with the LPS+Tria group. (I–K) HT-22 cells were treated with PBS (Ctrl), CM, and CM cotreated with 1 μM Tria. (I) Representative fluorescent images of LDs visualized with BODIPY. Scale bars: 2.5 μm. (J) Average area of LDs (μm^2^) and (K) average number of LDs (per cell). Values are mean ± SEM, n = 3. ∗*P* < 0.05, ns, nonsignificant. CM, conditioned medium; LD, lipid droplet; LPS, lipopolysaccharide.

LDs contain neutral lipids, such as TAGs and CEs, with their composition varying between cell types (27). Our study revealed elevated levels of TAG and CE in microglial cells treated with LPS (Fig. 4A, B). However, the concentration of TAGs and CEs in the supernatant of LPS-stimulated microglia did not show a significant increase (*P* < 0.05) (Fig. 4C, D). This finding suggests that LD produced by microglia are not released into the supernatant and subsequently transferred to neurons. Triacsin C, a native intracellular long-chain acyl-CoA synthetase inhibitor, blocks TAG accumulation into LD (29). Blocking lipid synthesis in the CM group did not restore neuronal viability (Fig. 4E). Moreover, the treatment with triacsin C, resulted in a significant decrease (*P* < 0.05) in LD accumulation in microglia by inhibiting TAG synthesis (Fig. 4F–H). However, the inhibition of TAG synthesis with triacsin C did not alter the LDs' area or number in the CM group (Fig. 4I–K).

### Activated microglia induce neuronal LD aggregation through lactic acid signaling

**Fig. 5:**
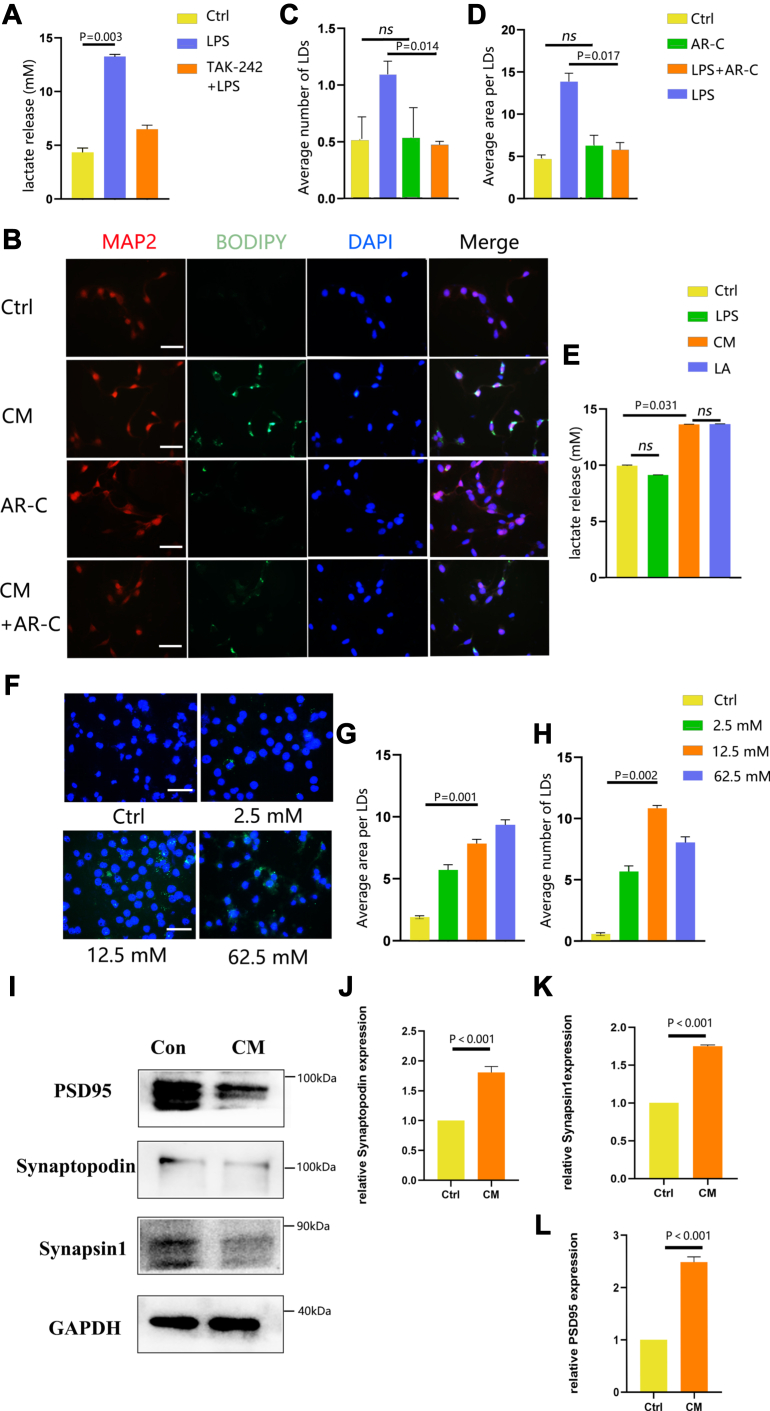
Neuronal LD aggregation is induced by lactic acid. (A) Measurement of lactic acid levels in the supernatant of BV2 cells by colorimetry. (B–D) Transwell microglia-neuron coculture system treated with CM or PBS and the AR-C155858 (AR-C). Upon AR-C treatment, LD aggregation was decreased in neurons. (C) Average number of LDs (per cell) and (D) average area of LDs (μm^2^). (E) Measurement of lactic acid levels in the supernatant of HT22 cells by colorimetry. (F–H) Neuronal stimulation with different concentrations of lactic acid. (F) Representative fluorescent images of LDs visualized with BODIPY. (G) Average area of LDs (μm2) and (H) average number of LDs (per cell). (I) Representative Western blot images. GAPDH was used as a loading control. (J–L) Band density of caspase1 represented as values quantified from protein bands of PSD95, synapsin1, and synaptopodin normalized to GAPDH. Values are mean ± SEM, n = 3. ∗*P* < 0.05; and ∗∗*P* < 0.01. Scale bars: 10 μm. CM, conditioned medium; LD, lipid droplet.

To identify the potential secreted mediators involved in microglia-induced LD aggregation in neurons during inflammation, we hypothesized that, in an inflammatory state, microglia activate the downstream signaling pathway of TLR4, leading to aerobic glycolysis (30). Lactic acid, a primary metabolite of aerobic glycolysis, is likely to play an essential role in this process. Therefore, to explore how microglia regulate LDs aggregation in neurons, we measured the lactic acid content in microglia. The lactate concentration in the supernatant of untreated microglia was 4 uM. We observed a significant increase (*P* < 0.05) in lactic acid content in microglial supernatants after LPS stimulation compared to control, reaching 12.5 uM (Fig. 5A). In contrast, the lactic acid content decreased in microglial supernatants after TAK-24, the TLR4 blocker, stimulation compared to the control (Fig. 5A). Further experiments involved treating HT-22 cells with CM after pretreatment with AR-C155858, an inhibitor of MCT1 and MCT2. We observed a significant decrease (*P* < 0.05) in both the average area and the number of LDs, suggesting that lactic acid may indeed play a crucial role in regulating LD formation. Subsequently, when we treated neurons directly with LPS, we found that LPS stimulation alone did not induce LD aggregation (Fig. 5B–D). Furthermore, LPS stimulation did not result in any changes in the lactic acid content of neurons (Fig. 5E). This suggests that neurons do not autonomously generate the accumulation of LDs but are regulated by microglial-derived lactic acid. To confirm this, we directly added 12.5uM lactic acid to the neurons (consistent with the lactate content produced by LPS stimulation of microglia), resulting in a significant accumulation (*P* < 0.05) of LDs in neurons (Fig. 5F–H). Synapses are the critical sites for information transmission between neurons. Under the influence of neuronal activities, synapses can undergo specific structural and functional changes to conduct nerve impulses, a phenomenon known as synaptic plasticity (31). Neuronal synaptic plasticity plays a vital role in the learning and memory functions of the nervous system. Proteins associated with synaptic plasticity are essential for maintaining the normal synaptic structure and information transmission functions of neurons. Therefore, we detected the expression of neuronal synaptic plasticity-related proteins, including postsynaptic density protein 95, synapsin1, and synaptopodin. Western blot results showed that after the addition of the activation of microglial supernatant, the expression of neuronal synaptic plasticity-related proteins was significantly downregulated (Fig. 5I–L). This indicates that microglial supernatant may inhibit the expression of neuronal synaptic plasticity-related proteins, causing impairment of synaptic plasticity functions in neurons, and ultimately leading to damage in cognitive functions such as learning and memory.

### Microglia regulate neuronal LD aggregation in an in vivo neuroinflammation model

**Fig. 6:**
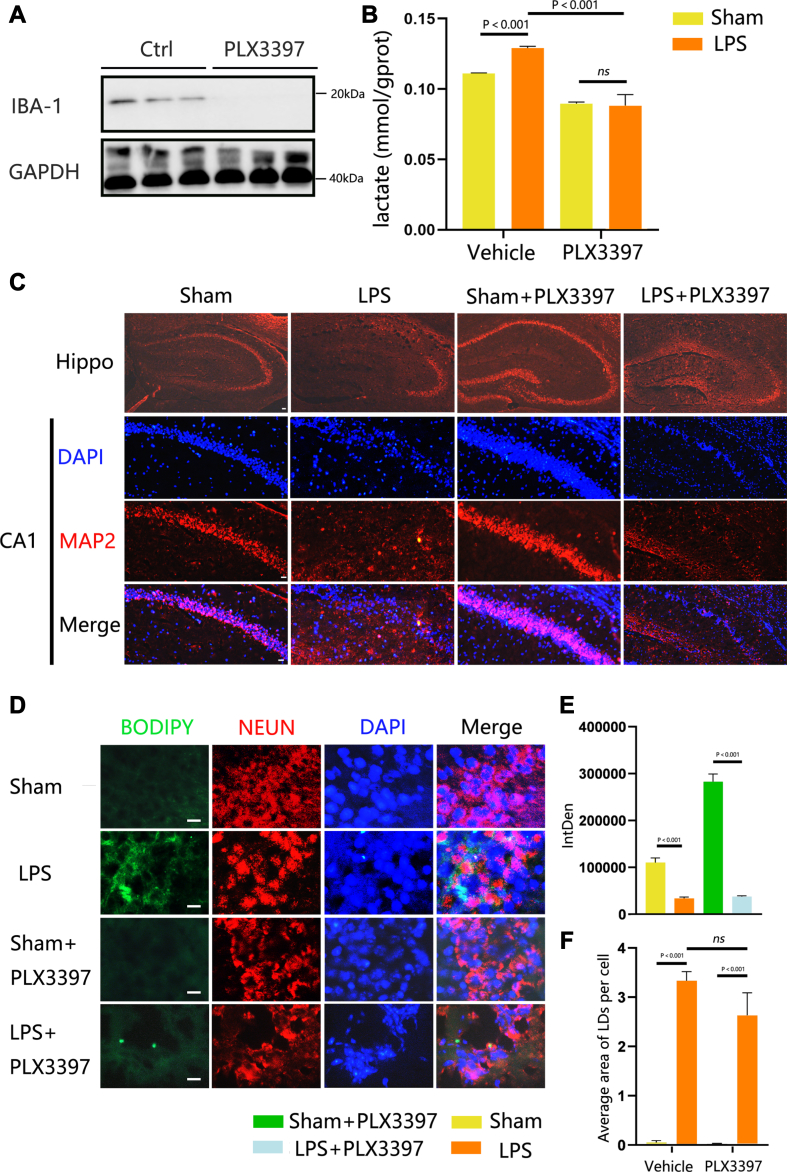
Regulation of neuronal LD aggregation by microglia in the neuroinflammation mouse model. Representative Western blot images of IBA-1 levels. GAPDH was used as a loading control. (B) Measurement of lactate levels in the brain using colorimetry. (C) Brain sections were stained with MAP-2 to visualize neurons and DAPI to visualize nuclei. (D) Brain sections were stained with BODIPY to visualize LDs, NEUN to visualize neurons, and DAPI to visualize nuclei. (E) Band density of MAP-2 is represented as values quantified from protein bands of MAP-2 normalized to GAPDH. (F) Average area of LDs per neuron cell. Values are mean ± SEM, n = 4, Scale bars: 100 μm, 50 μm, and10 μm, respectively. BA1, ionized calcium-binding adapter molecule 1; DAPI, 4',6-diamidino-2-phenylindole; IMAP-2, microtubule-associated protein-2; LD, lipid droplet.

Given the observation that activated microglia induced the accumulation of LDs in HT22 cells, we further investigated the involvement of microglia-mediated LD aggregation in a neuroinflammation mouse model. Neuroinflammation, encompassing diverse inflammatory events in the CNS, occurs in response to various pathological conditions such as infection, trauma, ischemia, and toxins (32). Moreover, systemic inflammation resulting from infection and sepsis can cause substantial alterations in cerebral homeostasis (33). Therefore, we utilized a mouse neuroinflammation model to analyze these alterations in an in vivo neuroinflammation setting. We treated both sham and LPS-treated mice with PLX3397, a colony-stimulating factor 1 receptor inhibitor or vehicle (34). PLX3397 had a strong inhibitory effect on microglia, leading to microglial death, as demonstrated by a substantial decrease in IBA-1 levels evident in the Western blot images (Fig. 6A). A significant increase (*P* < 0.05) in brain lactic acid concentration following neuroinflammation was observed, and this increase was significantly reduced (*P* < 0.001) after PLX3397 treatment in both the sham and LPS groups (Fig. 6B). Evaluation of hippocampal structure and neuronal density using MAP-2, NEUN, and BODYPI staining revealed damaged hippocampal structure and decreased neuron count in the neuroinflammation group compared to the sham group (Fig. 6C). Concurrently, we observed a significant increase (*P* < 0.01) in LD accumulation in the LPS group compared to the sham group, and this LD accumulation was significantly mitigated in the PLX3397-treated neuroinflammation group compared to the neuroinflammation group (Fig. 6D, E). These findings indicate that microglia play a pivotal role in mediating LDs aggregation during neuroinflammation. Furthermore, the observed increase in lactic acid content suggests that microglia may influence LD accumulation through the lactic acid pathway.

### Lactic acid induces LD accumulation through upregulated lipid metabolism

**Fig. 7:**
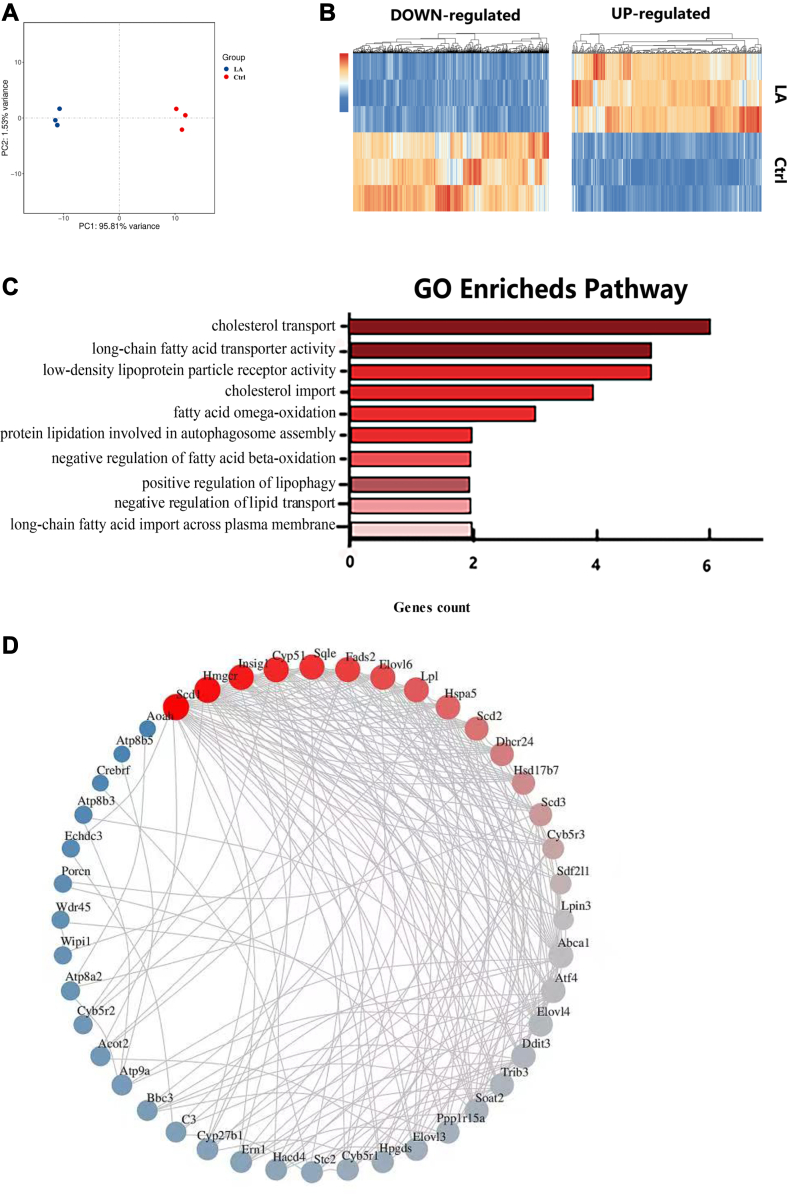
RNA sequencing of HT-22 cells revealed lactic acid–mediated regulation of genes and pathways influencing LD aggregation. HT-22 cells were incubated with lactic acid for 18 h, followed by transcriptome sequencing. (A) PCA plots from the whole transcriptome comparing the lactic acid–treated group with the control group. (B) Heat map displaying the differential expression of genes influenced by lactate compared to control cells. Transcript expression levels (count per million) are represented, with z-scores indicating the normalized expression of each transcript across the different conditions. (C) GO pathways overrepresentation analyses. Significantly enriched pathways (*P* < 0.05) are ranked based on genes count. (D) Protein-protein interaction networks between the lactate and control groups in each dataset. Red indicates upregulated gene expression; blue indicates downregulated gene expression. Genes with *P* < 0.05 are highlighted. GO, Gene Ontology; LD, lipid droplet; PCA, principal component analysis.

Lactic acid is recognized as a substrate influencing lipid metabolism. To comprehensively understand the additional impact of lactic acid, we conducted whole transcriptome RNA-seq, analyzing the expression of over 15,000 genes. We first validated the reproducibility and differences between the lactic acid group and the control group through principal component analysis (Fig. 7A). Treatment with lactic acid resulted in notable changes in the expression of 1,801 genes (386 upregulated and 1,415 downregulated) compared to the control group (Fig. 7B). Our sequencing data further highlighted the significance of the lipid metabolism pathway in the GO analysis (Fig. 7C). Overrepresentation analysis of GO pathways for the genes regulated by lactate revealed the involvement of cholesterol transport and long-chain FA transporter activity pathways. Additionally, further insight into the potential genes involved in lactic acid–mediated regulation of LD formation was gained through protein-protein interaction networks (Fig. 7D). Specifically, key proteins associated with cholesterol and FA metabolism, such as recombinant early growth response protein 1, myelocytomatosis proteins, squalene epoxidase (*Sqle*), and fatty acid desaturase 2 (*Fads2*), emerged as potential contributors to LD formation. *Sqle* serves as a rate-limiting enzyme for CE synthesis, while *Fads2* plays a crucial role in the conversion from saturated to unsaturated FAs. Additionally, we observed changes in the nuclear receptor subfamily 4, which is transcription factor of *Sqle.* This provides us with a hypothesis: whether lactic acid can affect LD formation by affecting nuclear receptor subfamily 4 and thereby regulating Sqle.

## Discussion

This study identified lactate as a critical mediator in the crosstalk between microglia and neurons, ultimately leading to LD aggregation in neurons and consequential functional impairment. Our findings provide compelling evidence highlighting the pivotal role of microglia in regulating LD aggregation in neurons, particularly under inflammatory conditions. Under normal physiological conditions, bidirectional neuron-microglia communication via the secretion of soluble factors regulates the functions of these cells. However, in the state of neuroinflammation, this communication becomes disrupted. Microglia undergo a metabolic shift from the oxidative phosphorylation pathway to glycolysis, releasing proinflammatory factors and substantial transcriptional changes in metabolic-related genes. This alteration leads to an increased expression of surface receptors and chronic secretion of cytokines, such as CCL3 and CCL4 (35). However, among the focus on proinflammatory factors, the significant changes in the products of glucose metabolism during inflammation are often overlooked. Therefore, we infer that under neuroinflammation, the increased production of lactic acid by microglia is transported to neurons through the microglia-neuron axis. This leads to the accumulation of LD in neurons, an increase in the expression of proteins indicative of cell death, and an enhanced cholesterol synthesis, which in turn causes LD aggregation in neurons.

Lactic acid is a highly glycolytic metabolite that can be transported to neighboring cells and oxidized to pyruvate as a primary energy source (36, 37). The transport of lactic between cells relies on MCTs, with MCT1, MCT2, and MCT4 being the most abundant in the brain (38). MCT1 is mainly expressed by endothelial, ependymal, and glial cells, while MCT4 by glial cells and MCT2 almost exclusively by neurons (39). Increased lactate levels not only impair the formation of hippocampal neurons in AD patients (40) but are also associated with apoptosis of cerebellar granule cells in early-stage AD patients (41). Additionally, lactate can promote the accumulation of α-synuclein and the apoptosis of dopaminergic neurons (12).

LD are typically found only in a few specific brain cells and do not aggregate in neurons; however, the abnormal accumulation of LDs in neurons under pathological conditions is often associated with a poor prognosis (42). Our study demonstrated abnormal LD accumulation in neurons incubated with CM from activated microglia, which was reversed by treatment with AR-C155858, indicating the dependence of lactic acid transporters for this effect. This suggests that lactic acid from activated microglia is transported to neurons through MCT. Notably, we observed that direct stimulation of neurons with LPS alone did not increase LDs number or area, suggesting LD production is not autonomously regulated by neurons but is influenced by other signaling molecules under inflammatory conditions.

Furthermore, lactic acid can serve as a substrate to promote FFAs synthesis, and excessive FFAs in neurons are associated with lipid peroxide chain reactions and the production of reactive oxygen species, leading to lipotoxicity (43). To prevent lipotoxicity, the surplus FFAs are stored in LDs, and the accumulated LDs in microglia can impair normal cell function (27). Our results revealed that LD aggregation in neurons decreased cell viability and increased pyroptosis. Supporting these observations, RNA-seq revealed that the lipid metabolism pathway emerges as a key player in the impact of lactate on neuronal function. The genes identified in our protein-protein interaction networks suggest potential targets for lactate regulation. For instance, overexpression of Sqle has been associated with increased mRNA expression of cholesterol biosynthesis genes (44), suggesting that Sqle may stimulate de novo cholesterol biosynthesis. Additionally, FADS2, acting as the primary rate-limiting enzyme in polyunsaturated FAs biosynthesis, was positively correlated with elevated concentrations of FAs and has been demonstrated to promote LD accumulation in epithelial cells (45). These findings lay the groundwork for further exploration of the molecular pathways involved in lactate signaling through targeted interventions in subsequent experiments.

In the CNS, astrocytes are the primary producers of lactic acid, derived from glucose or glycogen, to meet the energetic demands of neurons and modulate their function (46). Previous studies have demonstrated that stimulation with lactic acid triggers LD accumulation in astrocytes (36); however, our study did not observe similar LD accumulation in neurons when activated astrocytes were present during neuroinflammation. Exposure to stress response, metabolic and hypoxic stress enormously facilitated LD accumulation in astrocytes (47). Nevertheless, astrocytes may be less sensitive to LPS stimulation concerning LD formation.

While scarce, previous reports on LD aggregation in neurons have shown conflicting results. Ji *et al.* reported a decrease in LD aggregation in SH-SY5Y cells following the incubation with BV2 cell line supernatant (48), which is inconsistent with our findings. We speculate that this discrepancy may stem from variations in experimental conditions, culture systems, such as the source and passage of cell lines, or differences in the LPS processing method. Despite the valuable insights gained from our study, we must acknowledge certain limitations. The CM derived from BV2 cells should be further validated using primary microglial cells for greater physiological relevance. Additionally, further confirmation of the effects of the CM in other animal models of neuroinflammation, such as rodent models of middle cerebral artery occlusion or cerebral edema, is warranted.

In conclusion, our study significantly advances our understanding of the role of lactic acid in inducing LD aggregation in neurons under the influence of activated microglia. These findings open avenues for future research, exploring the underlying mechanisms by targeting specific genes associated with neuronal LD aggregation. Such insights hold promise for the development of more effective treatments for neuroinflammatory diseases.

## Data availability

All relevant data are included in the article.

## Conflict of interests

The authors declare that they have no conflicts of interest with the contents of this article.

## References

[1] Candelario-Jalil E., Dijkhuizen R.M., Magnus T. (2022). Neuroinflammation, stroke, Blood-brain dysfunction, and imaging modalities. Stroke.

[2] Ma X.Z., Chen L.L., Qu L., Li H., Wang J., Song N. (2024). Gut microbiota-induced CXCL1 elevation triggers early neuroinflammation in the substantia nigra of Parkinsonian mice. Acta Pharmacol. Sin..

[3] Meiser J., Krämer L., Sapcariu S.C., Battello N., Ghelfi J., D'Herouel A.F. (2016). Pro-inflammatory macrophages sustain pyruvate oxidation through pyruvate dehydrogenase for the synthesis of itaconate and to enable cytokine expression. J. Biol. Chem..

[4] Lambertsen K.L., Finsen B., Clausen B.H. (2019). Post-stroke inflammation-target or tool for therapy?. Acta Neuropathol..

[5] Zhou X., Zhao R., Lv M., Xu X., Liu W., Li X. (2023). ACSL4 promotes microglia-mediated neuroinflammation by regulating lipid metabolism and VGLL4 expression. Brain Behav. Immun..

[6] Monsorno K., Buckinx A., Paolicelli R.C. (2022). Microglial metabolic flexibility: emerging roles for lactate. Trends Endocrinol. Metab..

[7] Bosch M., Sánchez-Álvarez M., Fajardo A., Kapetanovic R., Steiner B., Dutra F. (2020). Mammalian lipid droplets are innate immune hubs integrating cell metabolism and host defense. Science.

[8] Voloboueva L.A., Emery J.F., Sun X., Giffard R.G. (2013). Inflammatory response of microglial BV-2 cells includes a glycolytic shift and is modulated by mitochondrial glucose-regulated protein 75/mortalin. FEBS Lett..

[9] Nagy A.M., Fekete R., Horvath G., Koncsos G., Kriston C., Sebestyen A. (2018). Versatility of microglial bioenergetic machinery under starving conditions. Biochim. Biophys. Acta Bioenerg..

[10] Liu Y., Yang S., Cai E., Lin L., Zeng P., Nie B. (2020). Functions of lactate in the brain of rat with intracerebral hemorrhage evaluated with MRI/MRS and in vitro approaches. CNS Neurosci. Ther..

[11] Xu J., Ji T., Li G., Zhang H., Zheng Y., Li M. (2022). Lactate attenuates astrocytic inflammation by inhibiting ubiquitination and degradation of NDRG2 under oxygen-glucose deprivation conditions. J. Neuroinflammation.

[12] Li J., Chen L., Qin Q., Wang D., Zhao J., Gao H. (2022). Upregulated hexokinase 2 expression induces the apoptosis of dopaminergic neurons by promoting lactate production in Parkinson's disease. Neurobiol. Dis..

[13] Schirinzi T., Di Lazzaro G., Sancesario G.M., Summa S., Petrucci S., Colona V.L. (2020). Young-onset and late-onset Parkinson's disease exhibit a different profile of fluid biomarkers and clinical features. Neurobiol. Aging.

[14] Olzmann J.A., Carvalho P. (2019). Dynamics and functions of lipid droplets. Nat. Rev. Mol. Cell Biol..

[15] Zhang J., Liu Q. (2015). Cholesterol metabolism and homeostasis in the brain. Protein Cell.

[16] Fester L., Zhou L., Bütow A., Huber C., von Lossow R., Prange-Kiel J. (2009). Cholesterol-promoted synaptogenesis requires the conversion of cholesterol to estradiol in the hippocampus. Hippocampus.

[17] Espinosa G., López-Montero I., Monroy F., Langevin D. (2011). Shear rheology of lipid monolayers and insights on membrane fluidity. Proc. Natl. Acad. Sci. U. S. A..

[18] Madra M., Sturley S.L. (2010). Niemann-Pick type C pathogenesis and treatment: from statins to sugars. Clin. Lipidol..

[19] Block R.C., Dorsey E.R., Beck C.A., Brenna J.T., Shoulson I. (2010). Altered cholesterol and fatty acid metabolism in Huntington disease. J. Clin. Lipidol..

[20] Wang Q., Yan J., Chen X., Li J., Yang Y., Weng J. (2011). Statins: multiple neuroprotective mechanisms in neurodegenerative diseases. Exp. Neurol..

[21] Roh K., Noh J., Kim Y., Jang Y., Kim J., Choi H. (2023). Lysosomal control of senescence and inflammation through cholesterol partitioning. Nat. Metab..

[22] Li Y., Ran Q., Duan Q., Jin J., Wang Y., Yu L. (2024). 7-Dehydrocholesterol dictates ferroptosis sensitivity. Nature.

[23] Marzan D.E., Brügger-Verdon V., West B.L., Liddelow S., Samanta J., Salzer J.L. (2021). Activated microglia drive demyelination via CSF1R signaling. Glia.

[24] Wang J., Zhu Q., Wang Y., Peng J., Shao L., Li X. (2022). Irisin protects against sepsis-associated encephalopathy by suppressing ferroptosis via activation of the Nrf2/GPX4 signal axis. Free Radic. Biol. Med..

[25] Li M.-Y., Zhu X.L., Zhao B.X., Shi L., Wang W., Hu W. (2019). Adrenomedullin alleviates the pyroptosis of Leydig cells by promoting autophagy via the ROS-AMPK-mTOR axis. Cell Death Dis..

[26] Khatchadourian A., Bourque S.D., Richard V.R., Titorenko V.I., Maysinger D. (2012). Dynamics and regulation of lipid droplet formation in lipopolysaccharide (LPS)-stimulated microglia. Biochim. Biophys. Acta.

[27] Marschallinger J., Iram T., Zardeneta M., Lee S.E., Lehallier B., Haney M.S. (2020). Lipid-droplet-accumulating microglia represent a dysfunctional and proinflammatory state in the aging brain. Nat. Neurosci..

[28] Conte M., Medici V., Malagoli D., Chiariello A., Cirrincione A., Davin A. (2022). Expression pattern of perilipins in human brain during aging and in Alzheimer's disease. Neuropathol. Appl. Neurobiol..

[29] Dechandt C.R.P., Zuccolotto-Dos-Reis F.H., Teodoro B.G., Fernandes A.M.A.P., Eberlin M.N., Kettelhut I.C. (2017). Triacsin C reduces lipid droplet formation and induces mitochondrial biogenesis in primary rat hepatocytes. J. Bioenerg. Biomembr..

[30] Li R., Yang Y., Wang H., Zhang T., Duan F., Wu K. (2023). Lactate and lactylation in the brain: Current progress and perspectives. Cell Mol. Neurobiol..

[31] Magee J.C., Grienberger C. (2020). Synaptic plasticity forms and functions. Annu. Rev. Neurosci..

[32] Leng F., Edison P. (2021). Neuroinflammation and microglial activation in Alzheimer disease: where do we go from here?. Nat. Rev. Neurol..

[33] Tauber S.C., Djukic M., Gossner J., Eiffert H., Brück W., Nau R. (2021). Sepsis-associated encephalopathy and septic encephalitis: an update. Expert Rev. Anti-infective Ther..

[34] Wang X., Fan W., Li N., Ma Y., Yao M., Wang G. (2023). YY1 lactylation in microglia promotes angiogenesis through transcription activation-mediated upregulation of FGF2. Genome Biol..

[35] Tauffenberger A., Fiumelli H., Almustafa S., Magistretti P.J. (2019). Lactate and pyruvate promote oxidative stress resistance through hormetic ROS signaling. Cell Death Dis..

[36] Horvat A., Zorec R., Vardjan N. (2021). Lactate as an astroglial signal augmenting aerobic glycolysis and lipid metabolism. Front. Physiol..

[37] Brooks G.A. (2009). Cell-cell and intracellular lactate shuttles. J. Physiol..

[38] Payen V.L., Mina E., Van Hée V.F., Porporato P.E., Sonveaux P. (2020). Monocarboxylate transporters in cancer. Mol. Metab..

[39] Kong L., Wang Z., Liang X., Wang Y., Gao L., Ma C. (2019). Monocarboxylate transporter 1 promotes classical microglial activation and pro-inflammatory effect via 6-phosphofructo-2-kinase/fructose-2, 6-biphosphatase 3. J. Neuroinflammation.

[40] Pol A., Gross S.P., Parton R.G. (2014). Review: biogenesis of the multifunctional lipid droplet: lipids, proteins, and sites. J. Cell Biol..

[41] Scorletti E., Carr R.M. (2022). A new perspective on NAFLD: focusing on lipid droplets. J. Hepatol..

[42] Chang H., Di T., Wang Y., Zeng X., Li G., Wan Q. (2019). Seipin deletion in mice enhances phosphorylation and aggregation of tau protein through reduced neuronal PPARγ and insulin resistance. Neurobiol. Dis..

[43] Ioannou M.S., Jackson J., Sheu S.H., Chang C.L., Weigel A.V., Liu H. (2019). Neuron-astrocyte metabolic coupling protects against activity-induced fatty acid toxicity. Cell.

[44] Li C., Wang Y., Liu D., Wong C.C., Coker O.O., Zhang X. (2022). Squalene epoxidase drives cancer cell proliferation and promotes gut dysbiosis to accelerate colorectal carcinogenesis. Gut.

[45] Wu J., Luo J., Xia Y., An X., Guo P., He Q. (2023). Goat FADS2 controlling fatty acid metabolism is directly regulated by SREBP1 in mammary epithelial cells. J. Anim. Sci..

[46] Magistretti P.J., Allaman I. (2018). Lactate in the brain: from metabolic end-product to signalling molecule. Nat. Rev. Neurosci..

[47] Smolič T., Tavčar P., Horvat A., Černe U., Halužan Vasle A., Tratnjek L. (2021). Astrocytes in stress accumulate lipid droplets. Glia.

[48] Ji Y., Wang X., Kalicki C., Menta B.W., Baumgardner M., Koppel S.J. (2019). Effects of microglial cytokines on Alzheimer's disease-related phenomena. J. Alzheimer's Dis..

